# A Note on Excess Mortality Attributable to COVID-19 in the United States

**DOI:** 10.31586/gjeid.2021.164

**Published:** 2021-11-10

**Authors:** James A. Koziol, Jan E. Schnitzer

**Affiliations:** Proteogenomics Research Institute for Systems Medicine, La Jolla, California, USA

**Keywords:** COVID-19 pandemic, Excess mortality, Difference in differences

## Abstract

**Background:**

Annual influenza outbreaks constitute a major public health concern in the United States. But this health burden appears dwarfed by the impact of COVID-19. Our aim is to quantify the excess mortality attributable to COVID-19, compared to previous influenza seasons.

**Methods:**

We retrospectively compare weekly mortality figures attributable to influenza and pneumonia in the United States from 2013 to 2019 with corresponding figures attributable to influenza, pneumonia, and COVID-19 from 2019 to 2021. We utilize a difference in differences regression methodology to estimate excess mortality observed in 2019–21 compared to 2013–2019.

**Results:**

Mortality patterns attributable to influenza, pneumonia, and COVID-19 differ significantly from the 2013–19 experience. Notably, distinct, aperiodic mortality waves occur in the 2019–2021 window, and mortality is well in excess of what is observed in typical influenza seasons.

**Conclusions:**

The COVID-19 pandemic has led to considerable excess mortality in the United States, and has strained public health resources. One might expect that the mortality waves observed during the pandemic will be damped by increasing levels of vaccination, and prior infections.

## 1. Introduction

The COVID-19 pandemic has led to over 700,000 deaths attributable to SARS-CoV-2 induced illness in the United States, far in excess of what might be expected in “typical” influenza seasons. The purpose of this note is to quantify the excess mortality associated with COVID-19, compared to previous influenza seasons. Our approach is through a difference in differences regression analysis, and is patterned after recent related studies [1,2].

## 2. Methods

Data on United States mortality attributable to influenza and pneumonia were obtained from the U.S. Centers for Disease Control website [3]. These data are weekly mortality totals attributable to influenza and pneumonia for the influenza seasons 2013–14 through 2020–21. For each season, the weekly mortality totals begin with calendar week 40 and continue through calendar week 39 of the subsequent year. For the last two seasons, namely 2019–20 and 2020–21, influenza and pneumonia deaths were combined with deaths attributable to COVID-19 (designated PIC hereafter). There were no deaths attributable to COVID-19 prior to the 2019–20 season; hence comparison of PIC mortality from 2019 through 2021 to influenza and pneumonia deaths prior to 2019 would delineate excess mortality attributable to COVID-19. Following Lee [1] and Sakamoto [2], we formulated a linear regression model for the weekly mortality data from 2013–14 through 2020–21, with categorical (fixed effect) predictors, namely, categorical variables for each week (1 through 52), an indicator variable (0–1) for SARS-CoV-2 exposure (0 for all weekly deaths prior to week 10 of 2020, then 1 for all deaths in subsequent weeks), and interaction variables corresponding to the week*exposure terms. Our focus here is not on the statistical significance of the regression results, rather, on the difference in differences estimates of excess deaths attributable to COVID-19, compared to previous years’ mortality solely attributable to influenza and pneumonia. All regressions were performed in Matlab v. R2017a.

## 3. Results

**Figure 1.**
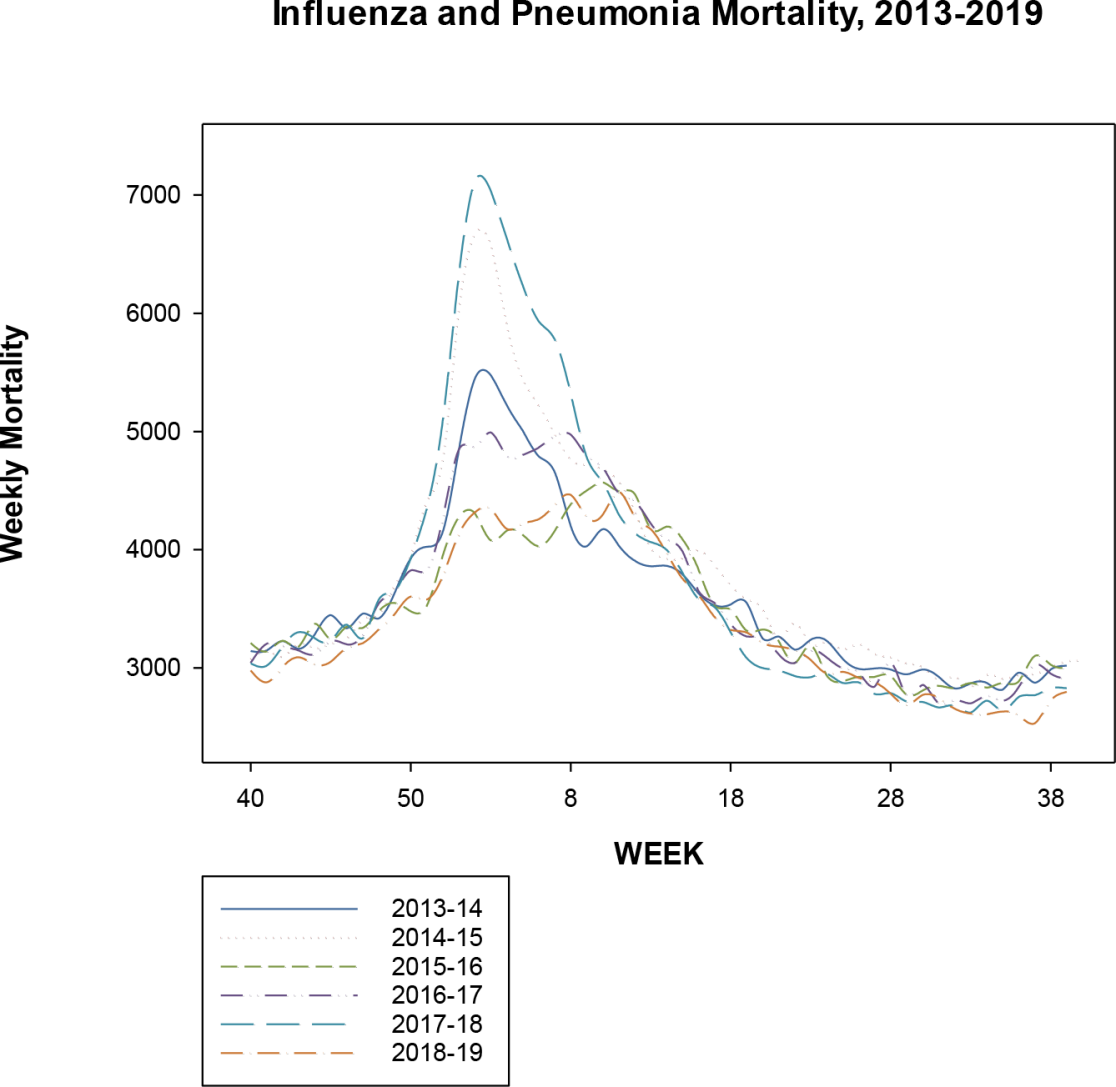
Weekly mortality attributable to influenza and pneumonia in the United States, for the annual influenza seasons 2013–14 through 2018–19. The Centers for Disease Control tabulations begin with week 40 of each initial year through week 39 of the following year.

**Figure 2.**
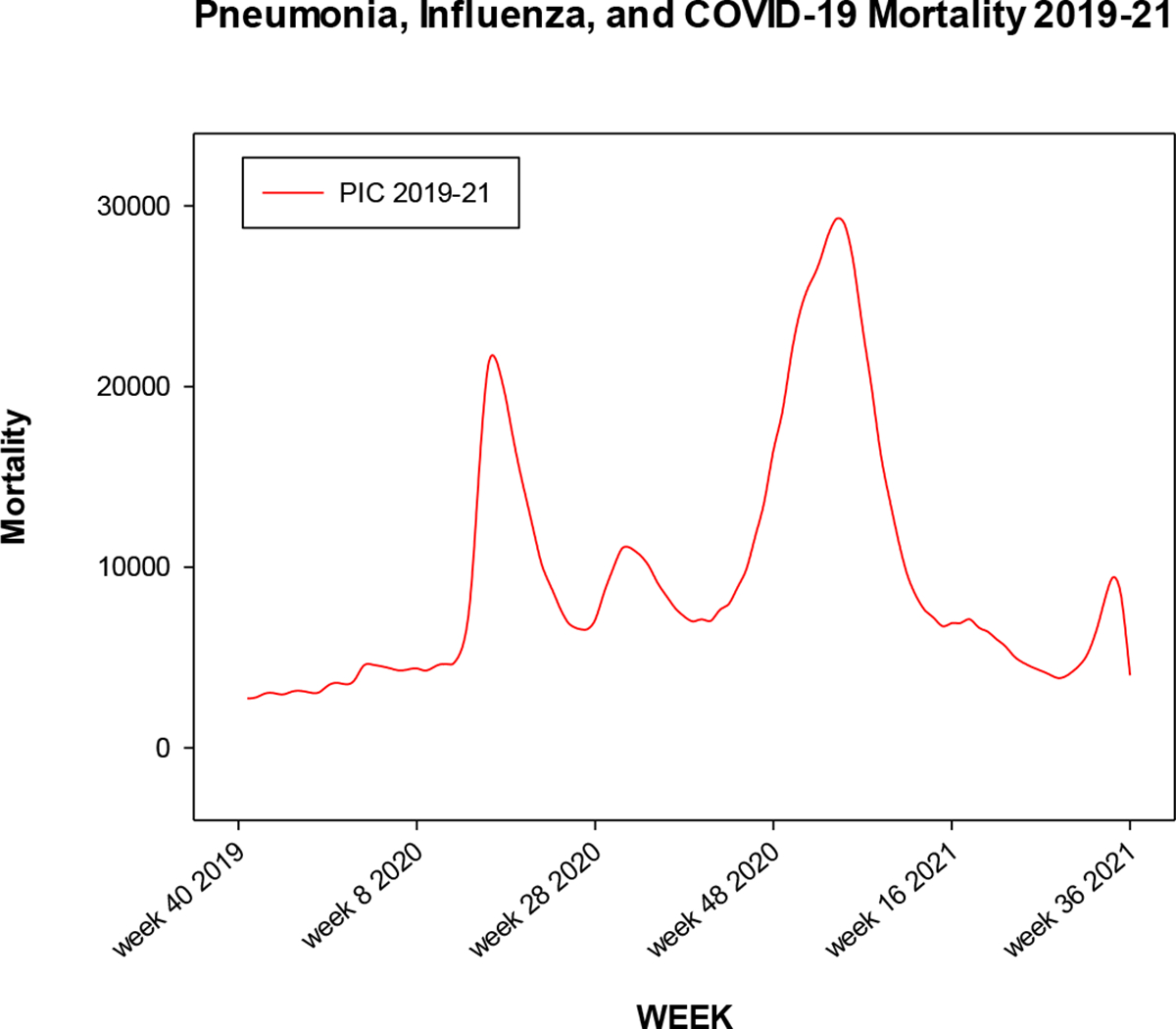
Weekly mortality attributable to influenza, pneumonia, and COVID-19 in the United States, for 2019–20 and 2020–21. Tabulations begin with week 40 of 2019 and end with week 39 of 2021.

We present a plot of the weekly mortality figures for influenza and pneumonia for the 2013–14 through 2018–19 influenza seasons in Figure 1. There is some year to year heterogeneity in mortality, but the unimodality, centered around weeks 1 through 10, is apparent. In contrast, Figure 2 shows the weekly mortality figures for pneumonia, influenza, and COVID-19 (PIC) in the United States, starting from week 40 of 2019. We observe waves of mortality, with much greater numbers than evinced in the previous influenza seasons.

**Figure 3.**
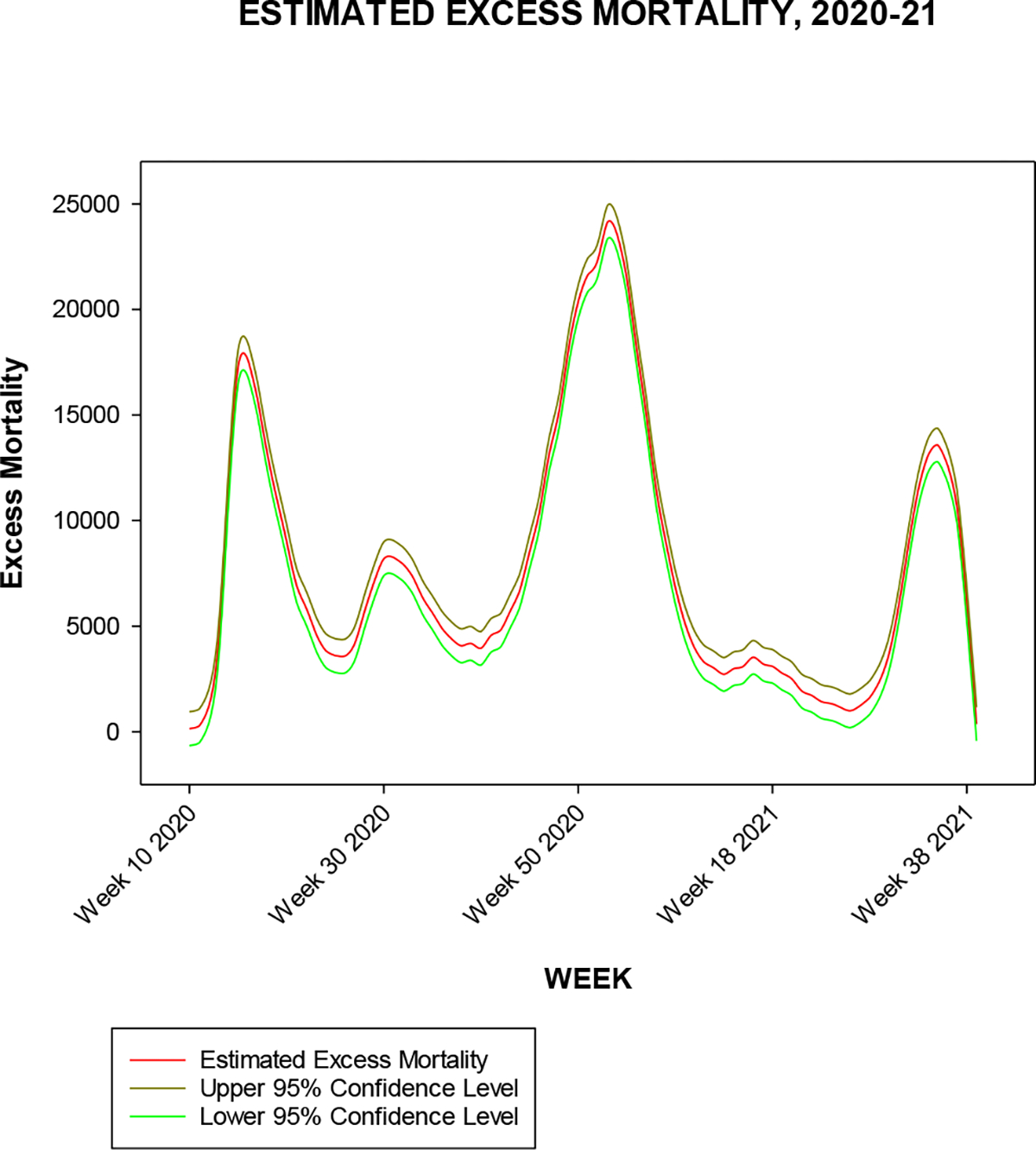
Estimated weekly excess mortality attributable to COVID-19 in the United States, from comparison of the 2019–21 experience (Figure 2) with the 2013–19 experience (Figure 1), with a difference in differences regression model. See text for further details.

In Figure 3 we show the difference in differences estimates of excess mortality attributable to PIC from week 10 of 2019 through week 39 of 2021, compared to the 2013 through 2019 experience, along with associated pointwise 95% confidence intervals. We remark that the widths of the confidence intervals are about 1600, though this appears minimal given the scaling of the mortality axis.

## 4. Discussion

It is clear from Figure 3 that PIC (pneumonia, influenza, and COVID-19) mortality far exceeds what would be expected in typical influenza seasons. But the figures highlight a unique feature of the COVID-19 pandemic in the United States compared to previous influenza seasons, namely, the occurrence of distinct waves of mortality, versus the unimodal mortality curves of previous influenza seasons. The waves are aperiodic, complicating modeling efforts, and do not follow the unimodal mortality pattern of previous influenza seasons. Furthermore, the waves appear somewhat attenuated with time, likely due to a reduced population susceptible to infection, as might be expected with increasing numbers of prior infections and rates of vaccination.

The difference in differences methodology originated in econometrics [4] and has seen widespread adoption in quantitative research studies utilizing observational data (e.g., ref. [5]). In the current setting, the relevant control vs. treatment comparison is between influenza and pneumonia mortality between 2013 and 2019 (with no exposure to SARS-CoV-2), and influenza, pneumonia, and COVID-19 mortality during 2019–21. The difference in differences methodology is meant to provide unbiased estimates of the differences in mortality attributable to SARS-CoV-2 induced illness, while mitigating the effects of biases due to extraneous factors, or trends.

A concern of any excess mortality determination is how to establish a baseline level of mortality for any subsequent comparison. We have previously addressed this issue [6], and approach this endeavor with caution, as statistical methodology may not altogether adequately account for trends and variability in mortality from season to season.

## References

[1] Lee H-H, Lin S-H. Effects of COVID-19 prevention measures on other common infections, Taiwan. Emerging Infectious Diseases 2020;26:2509–2511. 32730735 10.3201/eid2610.203193 PMC7510692

[2] Sakamoto H, Ishikane M, Ueda P. Seasonal influenza activity during the SARS-CoV-2 outbreak in Japan. JAMA 2020;323:1969–1971. 32275293 10.1001/jama.2020.6173 PMC7149351

[3] https://gis.cdc.gov/grasp/fluview/mortality.html

[4] Ashenfelter O, Card D. Using the longitudinal structure of earnings to estimate the effect of training programs. The Review of Economics and Statistics 1985;67,648–660.

[5] Card D, Krueger AB. (1994). Minimum wages and employment: A case study of the fast-food industry in New Jersey and Pennsylvania. American Economic Review 1994;84:772–793.

[6] Koziol JA, Schnitzer JE. Lessons from the past: Comparison of the disease burden of the Influenza A (H1N1) pandemic 2009–10 and seasonal influenza 2010–2019 in the United States. Journal of Infectious Diseases and Epidemiology 2021;7:218. 34988352 10.23937/2474-3658/1510218 PMC8725685

